# Understanding and Managing Sepsis in Patients With Cancer in the Era of Antimicrobial Resistance

**DOI:** 10.3389/fmed.2021.636547

**Published:** 2021-03-31

**Authors:** Carlota Gudiol, Adaia Albasanz-Puig, Guillermo Cuervo, Jordi Carratalà

**Affiliations:** ^1^Infectious Diseases Department, Bellvitge University Hospital, Bellvitge Biomedical Research Institute (IDIBELL), University of Barcelona, Barcelona, Spain; ^2^Institut Català d'Oncologia (ICO), Hospital Duran i Reynals, Bellvitge Biomedical Research Institute (IDIBELL), Barcelona, Spain; ^3^Spanish Network for Research in Infectious Diseases (REIPI RD16/0016/0001), Instituto de Salud Carlos III, Madrid, Spain

**Keywords:** sepsis, septic shock, cancer, bacteremia, bloodstream infection, neutropenia, antibiotic resistance

## Abstract

Sepsis is a frequent complication in immunosuppressed cancer patients and hematopoietic stem cell transplant recipients that is associated with high morbidity and mortality rates. The worldwide emergence of antimicrobial resistance is of special concern in this population because any delay in starting adequate empirical antibiotic therapy can lead to poor outcomes. In this review, we aim to address: (1) the mechanisms involved in the development of sepsis and septic shock in these patients; (2) the risk factors associated with a worse prognosis; (3) the impact of adequate initial empirical antibiotic therapy given the current era of widespread antimicrobial resistance; and (4) the optimal management of sepsis, including adequate and early source control of infection, optimized antibiotic use based on the pharmacokinetic and pharmacodynamics changes in these patients, and the role of the new available antibiotics.

## Introduction

Sepsis is defined as a life-threatening organ dysfunction caused by a dysregulated host response to infection. It is associated with significant morbidity and mortality (1–3) that increase markedly when septic shock becomes established. Moreover, sepsis and septic shock are major healthcare problems, affecting millions of people worldwide each year, with the increasing cost of sepsis-associated medical care now estimated at $17 billion annually in the United States (4). Given the rapidly expanding elderly population with their associated immune senescence and frailty (5), the mortality rates associated with sepsis are expected to increase dramatically over the next 2 decades (6).

The epidemiology of sepsis in industrialized countries is mainly influenced by the age of the population and the increasing prevalence of comorbidities, such as chronic organ dysfunctions, non-cancer-related immunosuppressive diseases, or cancer itself. Patients with cancer are at more than ten times higher risk for sepsis than the general population, with some variability according to the cancer types (7, 8). Mortality due to sepsis has decreased over time in these patients, probably due to improvements in the general management of sepsis, advances in cancer therapies, and improvements in the intensive care unit (ICU) admission policies. Nevertheless, in recent decades, we are facing the alarming emergence of antimicrobial resistance among microorganisms that cause infection and sepsis, in both the general population and the immunosuppressed alike, which can negatively influence outcomes (9). Of special concern is the widespread emergence and dissemination of multidrug-resistant (MDR) Gram-negative bacilli (GNB), which are a common cause of infection and sepsis in patients with cancer. Several investigators have reported high rates of bacteremia due to extended-spectrum β-lactamase (ESBL)-producing *Enterobacterales* (10–13), MDR *Pseudomonas aeruginosa* (MDR-PA) (12, 14–17), and carbapenem-resistant *Enterobacterales* (CRE) (18–22), among others, against which there are few treatment alternatives. This is of paramount importance because inadequate initial empirical antibiotic therapy can increase mortality when patients with cancer have infection due to MDR-GNB (11, 13–16, 18–23). Therefore, in the current era of widespread antibacterial resistance, there is an urgent need for the development of new agents with activity against MDR-GNB. In the meantime, novel ß-lactam/ß-lactamase inhibitors may be safe and effective options for treating infections due to some of these MDR-GNB (24, 25). In addition, specific strategies may help improve the overall prognosis of immunosuppressed patients with cancer, such as rapidly identifying sepsis (e.g., scores and biomarkers), optimizing ß-lactam antibiotic use (e.g., extended infusions), and optimizing source control and providing aggressive management in the ICU.

Finally, the pathophysiology of sepsis in the presence of cancer is especially complex because both entities share pathophysiological characteristics that result from the incapacity of the host's immune system to deal with an initial trigger. Thus, a dysregulated immune system seems common to both scenarios, raising the specter of their mutual impact on each other's course. Improving our knowledge about this bidirectional interaction between sepsis and cancer may lead to future research possibilities that could help modulate the dysfunctional immune system and the hyperinflammatory state, thereby improving sepsis control.

In this review, we aim to assess the prevalence, characteristics, etiology, and outcomes of sepsis in immunocompromised cancer patients and hematopoietic stem cell transplant (HSCT) recipients and to gain knowledge regarding the physiopathology of sepsis in these contexts. We also aim to review optimal management in the current era of widespread antimicrobial resistance. Finally, we will briefly comment on the current gaps in the literature and on directions for future research. Our focus is on sepsis due to bacterial infection. This review was not designed to provide evidence-qualified recommendations.

## Search Strategy and Selection Criteria

We searched PubMed/MEDLINE for articles that were published from January 2000 to October 2020, using the following terms: “sepsis,” “severe sepsis,” “septic shock,” “pathophysiology,” “immunosuppression,” “cancer,” “solid tumor,” “hematologic malignancy,” “hematopoietic stem cell transplant,” “neutropenia,” “bacteremia,” “bloodstream infection,” “intensive care unit,” “antibiotic resistance,” “multidrug resistance,” “mortality,” “SOFA,” “procalcitonin,” “C-reactive protein,” “adrenomedullin,” “ceftolozane/tazobactam,” “ceftazidime/avibactam,” and “extended infusion.” Articles resulting from these searches, together with any relevant references cited in those articles, were reviewed. We only included articles written in English and involving adult patients.

## The Burden of Sepsis in Cancer Patients

Some years ago, Angus *et al*. reported that one in 6 patients with sepsis presented a malignant underlying disease and that these patients suffered 30% excess mortality than other patients with sepsis (26). More recent ICU observational series have confirmed that about 15–20% of patients admitted to critical units have hematologic or solid malignancies (27–30), with sepsis being a leading cause of ICU admission in these patients (31, 32). Nevertheless, sepsis-associated mortality in cancer patients has decreased over recent decades (33–36), probably due to advances in sepsis diagnosis and management, cancer therapies, and ICU admission policies (37–39).

The current rates of in-hospital mortality of cancer patients presenting with sepsis and septic shock are ~20 and 40%, respectively (36). Sepsis-related mortality relies on not only appropriate early management of multiple organ failure but also minimizing prolonged ICU stays and associated complications (40, 41). Moreover, the long-term outcome of cancer sepsis survivors after ICU admission is depended on the prognostic determinants of the underlying diseases as well as the possibility of continuing antineoplastic treatment, which may be hampered by loss of functional status and/or persistent organ dysfunction (42–45). Importantly, since early sepsis mortality has decreased over time, attention has recently been paid to late mortality after recovery from sepsis. Even though the exact causes of long-term sepsis mortality are still unclear, some investigations suggest that older age, comorbidities, and persistent organ injury are detrimental and lead to the immune system's dysfunction and suppression, with persistent inflammation and catabolism (46, 47).

The increased risk of sepsis in cancer patients is due to several factors: immunosuppression caused by the underlying disease, the specific onco-hematological treatments causing immunosuppression, and the invasive procedures used (e.g., long-term central venous catheters, urinary catheters, drainages, etc.). However, the cancer population is heterogeneous and there is great variation in the degree of immunosuppression. Hematological patients are at highest risk of infection and sepsis, particularly those with acute leukemia who often present prolonged and profound neutropenia (36, 48, 49), historically one of the most important risk factors for sepsis and mortality (50, 51).

Multiple myeloma and HSCT also place patients at higher risk of sepsis compared with other hematological malignancies (36, 40). HSCT recipients represent a unique population that is severely immunosuppressed due to the underlying disorder, the conditioning regimen, and the treatment of complications, such as graft-vs. host-disease (GVHD). In this setting, allogeneic transplant recipients presenting with GVHD seem to be at higher risk of sepsis and death compared to non-HSCT recipients and both autologous and allogeneic patients without GVHD, reaching mortality rates as high as 55% (52). Among patients with solid tumors, the most sepsis occurs with lung or gastrointestinal cancers, followed by other subtypes depending on the series. In patients with solid malignancies, the site of primary or metastatic tumor often serves as the portal of entry (36, 48, 53, 54).

Even though febrile neutropenia (FN) is a frequent complication, occurring in 20–30% of patients with solid tumors and 80% of patients with hematological malignancies receiving chemotherapy, only 20–30% will develop bacteremia. Therefore, the rate of sepsis and septic shock globally is relatively low for FN. In line with this, a recent Brazilian study evaluating the frequency and epidemiology of early death and shock in 1,305 episodes of FN in 826 hematologic patients collected from 2003 to 2017 found that shock occurred in 42 (3.2%) on the first day of FN and early death occurred in 1.1% (55). In this study, predictors of septic shock were bacteremia due to *Escherichia coli* [odds ratio (OR), 8.47; 95% CI 4.08–17.55; *p* < 0.001), *Enterobacter sp*. (OR, 7.53; 95% CI 1.60–35.33; *p* = 0.01), and *Acinetobacter sp*. (OR, 6.95; 95% CI 1.49–32.36; *p* = 0.01).

## Pathophysiology of Sepsis in Cancer Patients

### Sepsis-Related Immunosuppression

Sepsis is an extremely complicated process in which several situations may occur and lead to a persistent immunosuppression and hyperinflammation. On the one hand, it induces a severe state of immunosuppression that affects both cellular effectors of the innate and adaptative immune systems, changes that can persist even after recovery (56, 57). These comprise functionally essential cells, such as neutrophils, monocytes and macrophages, natural killer (NK) cells, dendritic cells, B lymphocytes, and T lymphocytes (including gamma delta T cells, T_H_ cell subpopulations and regulatory T cells). On the other hand, sepsis induces a state of complex immune dysfunction, including hyperinflammation (excessive release of inflammatory cytokines IL-1, TNF, and IL-7) (58), homeostatic dysfunction (59), complement activation, fibrinolytic and clotting system stimulation (60), redox imbalance (causing severe oxidative stress) (61), mitochondrial dysfunction (62), and molecular alterations (causing organ damage) (56).

### Cancer-Related Immunosuppression

The immunosuppression in cancer that increases the risk of infection and sepsis is mainly associated with specific onco-hematological therapies that impair the immune system. Treatment with chemotherapy and radiotherapy alters the phagocytic activity of neutrophils and monocytes by depleting their circulating counts and impairing their capacity for chemotaxis and phagocytosis (63). As mentioned above, the risk of infection and sepsis is strongly associated with the depth and duration of neutropenia and monocytopenia (51, 52). Most cytostatics induce quantitative and/or functional modifications in lymphocytes and NK cells, while other anti-lymphoproliferative drugs and monoclonal antibodies (e.g., fludarabine, bendamustine, ibrutinib, rituximab, and alemtuzumab) can induce prolonged B- and/or T-cell lymphopenia (64). Corticosteroid use is also frequent in cancer patients, increasing the degree of immunosuppression. They cause both a pleiotropic dysregulation of innate and adaptive immune responses and a decrease in the activities of neutrophils, monocytes, macrophages, and lymphocytes (mainly CD4^+^ T cells). At high doses, they also induce apoptosis, decrease IL-2 levels, and impair the Th2-cell response. Therapy with HSCT may delay immune reconstitution due to persistent lymphopenia, low cell diversity, and defective lymphocyte functions (65). In addition, chemotherapy and radiotherapy may impair other organ and tissue functions, limiting their capacity to deal with the initial aggression. In this regard, it has been suggested that the endothelial toxicity secondary to cytostatic agents may lead to microcirculatory alterations and an impaired vessel response to vasopressors (66).

There are specific cancer settings that may increase the risk of infection and sepsis, regardless of antineoplastic therapy. The involvement of the bone marrow and/or the presence of dysmyelopoiesis by certain hematological malignancies or by metastatic solid tumors may lead to important cytopenias and/or defective phagocytic activity of neutrophils and monocytes (67). Some lymphoproliferative disorders may also cause hypogammaglobulinemia, and the compression of anatomic structures and/or tissue infiltration by malignant cells can diminish local defense mechanisms.

Finally, tumor cells can escape cytotoxic cells by losing major histocompatibility class-1 molecules, leading to the inappropriate expression of checkpoint inhibitory molecules, and can exhibit functional defects that result in decreased antigen presentation, and altered dendritic, macrophage, NK, and CD8 T cell function (68). Whether tumor-related immune alterations increase the risk of infection still needs to be confirmed.

### The Bidirectional Interaction Between Cancer and Sepsis

There are pathophysiological similarities between cancer and sepsis that favor the interaction between these two processes. Indeed, some malignancy-related conditions and adverse drug reactions can mimic sepsis (69) and may hinder differentiation between these entities. In particular, certain aggressive hematological diseases, such as acute leukemia and high-grade B-cell lymphoma may present multiple organ dysfunctions through several pathways, such as tissue infiltration by tumor cells, anatomical compression, intracellular metabolite release, altered coagulation, and hemophagocytic lymphohistiocytosis (70, 71). Currently available antineoplastic therapies, including T-cell based therapies (e.g., bispecific monoclonal antibodies and chimeric antigen receptor-T cells) or differentiating agents (e.g., all-trans retinoic arsenic or acid), may also produce acute systemic inflammatory syndromes that mimic sepsis (72, 73). Differentiating these entities is of paramount importance because managing these proinflammatory conditions varies significantly.

The similarities between cancer-related and sepsis-induced immune dysfunctions indicate that immune defects derived from infectious triggers may facilitate a favorable environment and promote tumor growth. Consistent epidemiological and experimental findings support a link between sepsis and further risk of cancer (74–77). Conversely, some historical reports and experimental studies have suggested that sepsis may instead have antitumoral activity (78, 79), with “cancer-then-sepsis” models suggesting that sepsis may induce tumor suppression (80–82).

Interestingly, recent data also suggest that antibiotic-induced dysbiosis (changes in the composition and diversity of gut microbiota) may alter the immune response to cancer (83, 84). Finally, it has been hypothesized that certain Toll-like receptors could have a role in modulating tumor growth in sepsis (85, 86).

## Characteristics, Etiology, and Outcomes of Sepsis in Cancer Patients

There are several current studies evaluating the prevalence, clinical features, etiology, and outcomes of cancer patients with bacteremia, with reported rates of septic shock varying by series from 6 to 57% (11, 13, 15, 18–20). Nevertheless, very few investigations have focused on patients presenting with sepsis or septic shock (31, 36, 48, 49, 52, 55, 87, 88), and among these, some were retrospective, some used different sepsis definitions, and some did not provide comprehensive data. Table 1 summarizes the most relevant data for cancer patients with sepsis or septic shock.

**Table 1:** Summary of the most relevant series of cancer patients presenting with sepsis and septic shock.

**Study**	**Rosolem et al. (31)**	**Torres et al. (87)**	**Lemiale et al. (36)**	**^c^Kumar et al.(**52**)**	
Study design	Secondary analysis of a prospective cohort study	Subgroup analysis of a multicenter prospective cohort study	Analysis of the GRRR-OH database which includes cancer patients from 1994 to 2015	Analysis of the healthcare cost and utilization project-nationwide inpatient sample (NIS) database	
Site	10-bed medical-surgical cancer ICU unit at Instituto Nacional de Cancer, Rio de Janeiro, Brazil	28 Brazilian ICUs	Seven European ICUs from France and Belgium	The database contains data from ~1,000 (20%) community U.S. hospitals	
Study period	January 2003–July 2007	August 1–September 2007	2006-2010	2000–2008	
Number of patients	563 patients with sepsis	268 patients with sepsis	2.062 patients with sepsis or septic shock	6.168 engraftment admissions in HSCT recipients with sepsis among 79.287 discharges (7.7%)	
				2.750 admissions for sepsis in autologous HSCT recipients (5.2%)	3418 admissions for sepsis in allogeneic HSCT recipients (13.2%)
Age (y)	59.2 ± 17.8	63.1 ± 15.0	59 (48–67)	21.6% (≥65 y)	4.35% (≥65 y)
Male sex	301 (54%)	126 (47%)	1.275 (61.8%)	56.6%	6.34%
Hematologic disease	127 (23%)	35 (13%)	1.700 (82.4%)	100%	100%
- NHL	14%	4%	461 (22.4%)	7.8%	3.2%
- Leukemia	6%	4%	591 (28.7%	16.9%	62.5%
- Multiple myeloma	2%	3%	244 (11.8%)	37%	4.5%
- Hodgkin lymphoma	–	–	–	10.4%	1.8%
HSCT recipients	–	8 (3%)	250 (14%)^a^	100%	100%
- Solid tumor	436 (77%)	233 (87%)	362 (17.6%)	–	–
- Gastrointestinal	35%	25%	61 (16.9%)	–	–
- Head and neck	13%	7%	4 (0.1%)	–	–
- Lung cancer	6%	9%	48 (13.3%)	–	–
-Urogenital	6%	15%	43 (11.8%)	–	–
-Breast	5%	6%	55 (15.2%)	–	–
- Uterus	–	–	15 (4.1%)	–	–
Recurrence/progression	103 18%	35%	–	–	–
PS >2	294 52%	57%	–	–	–
Neutropenia (<500/mm^3^)	71 13%	12%	640 (31%)	–	–
Comorbidities	349 (62%)	–	1.043 (50.6%)	–	–
-Diabetes mellitus	14%	–		6.8%	4.1%
-Hypertension	38%	–		–	–
-COPD	12%	–		11.8%	8.2%
Previous chemotherapy and/or radiotherapy	40%	54%	211 (58.3%)	–	–
**Acquisition**					
- Community-acquired	227 40%	32%	–	–	–
- Nosocomial	336 60%	67.5%	–	–	–
SOFA score (points) on the first day of ICU	8 (5–11)	9 (7–12)	6 (4–9)	–	–
Acute organ failures (n)	2 (1–3)	4 (3–4)	–	18% (≥3)	24.5% (≥3)
Mechanical ventilation	489 (87%)	51%	1.016 (49.3%)	30.9%	40.4%
Renal replacement therapy	110 (20%)	18%	420 (20.4%)	11.4%	19.3%
Vasopressor use	372 (64%)	59%	1.172 (56.8%)	–	–
**Proof of infection**					
- Clinically suspected	180 (32%)	133 (50%)	–	–	–
- Microbiologically proven	383 (68%)	135 (50.3%)			
**Severity**					
- Sepsis	48 (9%)	–	–	–	–
- Severe sepsis	143 (25%)	142 (53%)	–	–	–
- Septic shock	372 (66%)	126 (47%)	–	–	–
**Pathogens**					
*Gram-positive*	168 (30%)	34 (13%)	–	33%	38.3%
- Enterococci	92 (16%)	–	–	–	–
- *S. aureus*	61 (11%)	22 (8.2%)	–	–	–
- Group D streptococci	16 (3%)	–	–	–	–
- *S. pneumoniae*	13 (2%)	4 (1%)	–	–	–
- Other	12 (3%)	–	–	–	–
- CNS	297 53%)	13 (5%)	–	–	–
*Gram-negative*	91 16%	83 (31%)	–	16%	15.8%
- *Escherichia coli*	74 13%	27 (10%)	–	–	–
- *P. aeruginosa*	72 13%	29 (11%)	–	–	–
- *K. pneumoniae*	46 8%	27 (10%)	–	–	–
- *Enterobacter* spp	34 6%	–	–	–	–
- *Proteus* spp	18 4%	–	–	–	–
- *M. morganii*	14 3%	–	–	–	–
- *S. maltophilia*	92 16%	3 (1%)	–	–	–
- Other	43 8%	8 (2.9%)	–	–	–
*Fungi*			–	–	–
- *Candida* spp	35 6%	12 (4%)	–	3.3%	5.4%
- Other	8 1%	1 (1%)	–	2.2%^d^	5.8%^d^
Other infectious agents	19 (3%)	39 (15%)	–	12%^e^	13.4%^e^
Multidrug-resistant bacteria	81 14%	–	–	^−^	–
**Site of infection**					
Lung	246 44%	130 (48%)	–	–	–
Abdomen	172 31%	67 (25%)	–	–	–
Urinary tract	42 8%	45 (17%)	–	–	–
Skin/soft tissue	35 6%	26 (10%)	–	–	–
Primary bacteremia	24 4%	51 (19%)	–	–	–
Central nervous system	11 2%	3 (1%)	–	–	–
Surgical site infection	–	18 (7%)	–	–	–
Other/unknown	57 10%	18 (7%)	–	–	–
More than 1 site of infection	24 4%	71 (26%)	–	–	–
ICU LOS	9 (4–18)	7 (4–16)	–	–	–
Hospital LOS (days)	23 (11–43)	22 (13–38)	–	26 (21–37)	39 (30–58)
ICU mortality	289 51%	42%	–	–	–
In-hospital mortality	364 65%	56%	823 (39.9%)^b^	30.1%	55.1%

*NHL, Non-Hodgkin lymphoma; HSCT, hematopoietic stem cell transplant; COPD, chronic obstructive pulmonary disease; SOFA, Sequential Organ Failure Assessment; ICU, Intensive care unit; CNS, Coagulase-negative staphylococci; LOS, Length of hospital stay; GRRR-OH, Groupe de Recherché Respiratoire en Réanimation Onco-Hématologique*.

a *Only data on allogeneic HSCT were provided*;

b *Data on 30-day mortality were available in 943 patients (45.7%), approximated using hospital mortality in 879 patients (42.6%) and as last resort ICU mortality if the former were unavailable*;

c *Only data regarding engraftment admission are provided in this table. Data on subsequent admissions in HSCT recipients with and without graft-vs. host diseases are provided in the complete version of the manuscript*.

d *Infections due to Aspergillus spp.*;

e *Clostridioides difficile colitis*.

Overall, patients with hematological malignancies, mainly acute leukemia, are at greatest risk of sepsis (36, 48, 49). Patients with multiple myeloma (36, 48, 87) and non-Hodgkin lymphoma (31, 36, 55, 87) also seem to experience high rates of sepsis and septic shock. HSCT recipients have been included in only few series, but rates of sepsis range from 3 (87) to 12% (36), as reported in a recent large study of cancer with 2,062 patients admitted to seven European ICUs for sepsis or septic shock. In this study, data were extracted from the *Groupe de Recherche Respiratoire en Réanimation Onco-Hématologique* database, from 2006 to 2010. Patients more often had hematological (82.4%) than solid (17.6%) malignancies, and many (31%) had neutropenia at ICU admission. The 30-day mortality rate was 39.9% and decreased significantly over the study period [odds ratio (OR) 0.96; 95% CI, 0.93–0.98; *p* = 0.001].

Seeking to characterize the frequency and outcomes of sepsis in adult HSCT recipients and to compare them to non-transplant patients, Kumar et al. retrospectively analyzed a large nationwide administrative database from almost 20% of the community hospitals in the United States (52). Data were provided separately according to the reason for admission (engraftment admission or subsequent admission with and without GVHD). Of the 291,179 discharges with HSCT between 2000 and 2008, sepsis was identified in 21,898 (7.5%). The frequency of sepsis was 5 times higher in HSCT recipients than in non-transplant patients. Allogeneic transplant recipients and those with GVHD (10.4%) had significantly higher rates of sepsis than either autologous recipients (13.2 vs. 5.2%; *p* < 0.001) or those without GVHD (10.4 vs. 6.1%; *p* < 0.001). The unadjusted hospital-related mortality was significantly higher among allogeneic transplant recipients than non-HSCT recipients (55.1 vs. 32.9%, *p* < 0.001), but the mortality rates did not differ between autologous HSCT recipients and the non-transplant population. After adjustment, however, the odds of mortality were 3.81 times higher in allogeneic HSCT recipients (95% CI, 2.39–6.07) and 1.28 times higher in autologous recipients compared with non-transplant patients (95% CI, 1.06–1.53). Table 2 displays more detailed information on the engraftment admissions and compares patients according to the type of transplant.

**Table 2:** Risk factors associated with mortality in the most relevant series of sepsis and septic shock in cancer patients.

**References**	**Patients studied**	**Outcome**		**OR (95% CI)**	***P***
Rosolem et al. (31)	563 cancer patients with sepsis	In-hospital mortality	Admission to a medical ICU	2.19 (1.40–3.42)	0.001
			Active-newly diagnosed disease	1.76 (1.12–2.75)	0.013
			Active-recurrence/progression	2.42 (1.35–4.35)	0.003
			Performance status >2	3.57 (2.36–5.979)	<0.001
			Non urinary tract infection	3.28 (1.57–6.86)	0.002
			SIRS criteria ≥ 3	1.80 (1.20–2.72)	0.014
			Cardiovascular dysfunction	1.94 (1.27–2.94)	0.008
			Respiratory dysfunction	2.29 (1.24–4.23)	0.002
			Renal dysfunction	2.12 (1.34–3.35)	0.001
Torres et al. (87)	268 cancer patients with sepsis	In-hospital mortality	Organ dysfunction	1.48 (1.16–1.87)	0.001
			Hematological malignancy	2.57 (1.05–6.27)	0.038
			Performance status >2	2.53 (1.44–4.43)	0.001
			Polymicrobial infections	3.74 (1.52–9.21)	0.004
Lemiale et al. (36)	2.062 cancer patients with sepsis or septic shock	30-day mortality	Mechanical ventilation	3.25 (2.52–4.19)	<0.01
			Vasopressor use	1.42 (1.10–1.83)	<0.01
^a^Kumar et al. (52)	6.168 engraftment admissions in HSCT recipients with sepsis	Unadjusted in-hospital mortality	Allogeneic HSCT	2.12 (1.55–2.90)	NA
			Age 35–49 years	1.68 (1.08–2.60)	NA
			Age ≥65 years	2.08 (1.13–3.83)	NA
			Cirrhosis as comorbidity	4.49 (1.81–11.1)	NA
			Multiple myeloma	0.59 (0.40–0.89)	NA
			Respiratory failure	12.1 (8.64–16.8)	NA
			Cardiac failure	2.42 (1.59–3.66)	NA
			Renal failure	2.64 (1.95–3.56)	NA
			Metabolic failure	1.63 (1.07–2.49)	NA
			Hepatic failure	5.22 (2.29–11.8)	NA

a *Only data regarding engraftment admission are provided in this table. Data on subsequent admissions in HSCT recipients with and without graft-vs. host diseases are provided in the complete version of the manuscript*.

Among solid tumors, the rates of sepsis vary by the series, with gastrointestinal (31, 87) and lung cancers (48) being most susceptible. In a recent report of 1,009 patients with gynecological cancers receiving 10.239 cycles of chemotherapy, the incidence of septic shock during neutropenia was 3% and the mortality rate was 1.2% (88). The incidence of septic shock during neutropenia was also higher in patients older than 50 years (3.9 vs. 1.4%, *p* = 0.034), with a linear-by-linear association between the accumulated cycles of chemotherapy and the sepsis rate (*p* = 0.004). Also, patients who had received two or more courses of chemotherapy presented and increased incidence of neutropenic septic shock (NSS) compared with those receiving only one course (4.9 vs. 1.4%, *p* = 0.002). No significant differences were observed regarding the type of gynecological cancers and the status of the disease between patients with NSS and patients without NSS. The mortality rate of patients with NSS was 37.5%. In this study, the median age (64.0 vs. 56.5, *p* = 0.017) and the peak heart rate (149.5 vs. 123.5 min^−1^, *p* = 0.015) were significantly higher in the group of patients who subsequently died of NSS than in those who survived.

In ~30–50% of the sepsis and septic shock episodes occurring in patients with cancer, no microbiological diagnosis is achieved (31, 87). When a definitive microbiological diagnosis is made, blood cultures are the most useful tool because most episodes are secondary to bacteremia and/or high-inoculum infections, such as pneumonia, with frequent secondary dissemination to the bloodstream.

The site of infection is barely provided in the few series addressing sepsis in cancer patients. In a report addressing shock and early death in hematologic patients with FN, Rosolem et al. described the site of infection in 563 cases of sepsis (91% with septic shock) admitted in the ICU (31). The most frequent sites were the lung (44%), the abdomen (31%), and the urinary tract (8%), with 24 patients (4%) having more than one site. GNB were responsible for more than 50% of episodes, with *E. coli* (16%), *P. aeruginosa* (13%), and *Klebsiella pneumoniae* (13%) being most common. The overall ICU and hospital-related mortality were 51 and 65%, respectively; these were higher in patients with septic shock (62 and 74%) than in patients with either sepsis (36 and 55%) or sepsis (15 and 25%). Mortality rates were higher for patients with pneumonia and bacteremia than with gastrointestinal and urinary tract infections. End-of-life decisions were made for 29% of patients in their cohort.

In a subgroup analysis of a multicenter prospective cohort study in 28 Brazilian ICUs, 717 patients with cancer were analyzed (87). Among them, 37% had sepsis and 53% septic shock. The most frequent infection sites were the lungs (48%), abdomen (19%), bloodstream (primary) (19%), and urinary tract (17%). Half had a microbiologically confirmed infection, with GNB again being the most frequent cause (31%). ICU- and hospital-related mortality rates were 42 and 56%, respectively. End-of-life decisions were made in 17% of the patients.

## Risk Factors for Mortality in Cancer Patients With Sepsis and Septic Shock

Table 2 shows the risk factors associated with mortality for the most relevant series of patients with cancer who developed sepsis or septic shock. The most commonly variables associated with mortality are those related to the underlying disease (mainly hematological malignancies) (52, 87) and its status (uncontrolled cancer and poor performance status) (31, 87), the presence of one or more organ dysfunctions (31, 52, 87), and the need for organ support (36). Some other variables identified as risk factors for mortality include older age (52, 88), comorbidities (52), infection site (particularly pneumonia) (31), polymicrobial infection (87). Of note, the year of ICU admission has also been shown to influence cancer patient's outcomes significantly, with decreased mortality rates observed over time (36, 52). The nutritional status of cancer patients may also play an important role in the development of sepsis and on its outcomes. In this regard, a study conducted in our institution involving head and neck cancer patients (who are particularly malnourished) with bacteremia identified hypoalbuminemia as independent risk factor for bacteremia and for early and overall mortality (89). Importantly, inadequate initial empirical antibiotic therapy (IEAT) is widely recognized as an important risk factor for mortality in all patients, including those with immunosuppression due to cancer (14, 18, 23, 90–92). Kadri et al. recently published a retrospective cohort analysis of electronic health record data from 131 hospitals in the US that included 21,608 patients with bacteremia who received empirical antibiotics between 2005 and 2014 (93). Among them, 4,165 (19%) received IEAT, which was independently associated with increased mortality (adjusted OR 1.46; 95% CI, 1.28–1.66); *p* < 0.0001), regardless of whether sepsis or septic shock was present. Infection due to antibiotic-resistant organisms was strongly associated with an increased risk of receiving IEAT (adjusted OR 9.09; 95% CI 7.68–10.76; *p* < 0.0001).

Several studies have shown that cancer patients with infections due to resistant pathogens are more likely to receive IEAT (90–92). In addition, many of these studies have shown that failure to cover resistant organisms, and particularly MDR-GNB, significantly and independently impairs their outcomes (10, 11, 14, 18, 20, 22, 23). The presence of septic shock and/or the need for ICU admission have also frequently been identified as risk factors for mortality in cancer patients with bacteremia (94). Therefore, it is reasonable to hypothesize that these factors (IEAT and sepsis/septic shock) synergize to affect the prognosis of these patients negatively. However, there remains no firm evidence of how IEAT impairs outcomes in cancer patients with sepsis.

## Optimal Management of Sepsis in Cancer Patients

### Early Recognition and Diagnosis

In 2017, Rhodes et al. published the “Surviving Sepsis Campaign,” which are international guidelines for the management of adult patients with sepsis and septic shock. These guidelines provide the best evidence-based recommendations for the management of this life-threatening condition (95).

Several studies in the general population have demonstrated that the Sequential Organ Failure Assessment (SOFA) score better predicts hospital mortality for ICU patients with infection compared with the systemic inflammatory response syndrome. Therefore, a new definition of sepsis and septic shock was adopted in 2016 (1), and later studies have demonstrated that the new definitions are applicable to cancer with the same reliability as in the general population (94). In addition, the quickSOFA (qSOFA), constitutes a simple bedside clinical score that can be rapidly applied and allows the prompt identification of patients at greatest risk of need for admission to an intensive care unit. The qSOFA score is based in 3 simple variables, which include respiratory rate ≥22/min, alteration in mental status, and systolic blood pressure ≤ 100 mm Hg (96). The SOFA and qSOFA scores are therefore useful tools to identify and predict complications and mortality in these patients (97–99), and as such, should be applied to all cancer patients with suspected infection.

In recent decades, over 180 biomarkers have been evaluated as unsuitable for the diagnosis and prognosis of sepsis, being lactate one of the most frequently used (100). To date, none have demonstrated sufficient specificity or sensitivity for reasonable utility in clinical practice. Procalcitonin (PCT) and C-reactive protein (CRP) have perhaps been the most widely used, but they have limited ability to distinguish sepsis from other inflammatory conditions or to predict outcomes. More recently, elevated serum lactate has been used as a biomarker in the diagnosis of septic shock (1). There is also interest in the role of these biomarkers as diagnostic, prognostic, and theragnostic markers in febrile cancer patients, particularly those with FN. Despite the paucity of data about biomarkers in cancer patients, some reports have evaluated their role. In this regard, PCT has been shown to have better accuracy than CRP and IL-6 in differentiating infectious from non-infectious causes of fever in a meta-analysis of 27 studies in adult and pediatric cohorts (101). However, this meta-analysis included different types of underlying disease and evaluated different outcomes. Several other reports have shown that PCT can predict bacteremia in cancer patients with and without neutropenia, particularly those with infection by GNB, and that it may predict a need for organ support (102–106).

Adrenomedullin (ADM) is also elevated in sepsis, which results in pro-ADM that is present at higher levels in patients with localized infections and bacteremia than in healthy controls (107). Pro-ADM has shown to be more suggestive of sepsis than PCT in cancer patients (108), and its levels are more significantly elevated in patients with hematological cancers and localized infections than in those with no infections (108). Nevertheless, another study of critically ill cancer patient revealed that pro-ADM and PCT had similar areas under the roc curve for identifying bacteremia, both being superior to that of CRP (109). Other biomarkers, such as presepsin, IL-6, and IL-8, appear to be less useful (110). The role the currently available biomarkers in cancer patients with sepsis clearly needs to be elucidated further.

### Antibiotic Therapy

In the current era of growing antimicrobial resistance, the following general considerations need to be assessed before deciding on empirical antibiotic therapy in cancer patients (particularly in FN): prior history of colonization/infection with resistant pathogens; the presence of other risk factors for antibiotic resistance; the local epidemiology and resistance patterns in that hospital, unit, and geographical area; and other patient-related factors that may predict a complicated clinical course (e.g., older age, comorbidities, localized infection, and shock).

After this evaluation, cancer patients with sepsis or septic shock need urgent therapy with a broad-spectrum anti-pseudomonal BLA with or without other agents that are active both against the suspected organisms and at the site of infection (111). It remains controversial whether adding a short-course aminoglycoside to a broad-spectrum BLA regimen can benefit severely ill patients. An important meta-analysis (112), as well as a recent prospective observational cohort study of 648 ICU patients (113), failed to show this association. In the study by Ong et al. there was no association with a faster reversal of shock or an increased 14-day survival; however, that study mainly included immunocompetent patients, and only 4% received IEAT, probably due to low local levels of antibiotic resistance (113).

Interestingly, in a previously published study we observed improved early (7- and 14-day) mortality rates in those who received initial combination therapy, who also presented more frequently with septic shock. In a prospective study of 510 hospitalized patients with bacteremia in the context of neutropenia due to hematological malignancy, we also observed better 30-day survival in those who received combination therapy (94). Similar findings have been reported in the general population by other investigators (114, 115). Therefore, while awaiting the results of well-designed randomized clinical trials, we advocate the inclusion of short-course aminoglycoside therapy with a BLA for IEAT when treating neutropenic cancer patients in centers with a high prevalence of multidrug resistance, especially if sepsis or septic shock are present.

The empirical use of the two novel antibiotics, ceftolozane/tazobactam and ceftazidime/avibactam, should be considered in cancer patients at risk of infection due to MDR-GNB (e.g., MDR-PA, CRE, or ESBL-*Enterobacterales*), particularly if they present with sepsis or septic shock. Targeted therapy with these agents should be also considered if other first-line antibiotics are not viable treatment options because of a lack of activity or a high-risk of toxicity. Although these novel antibiotics have not been specifically approved for neutropenic and/or cancer patients, they are being used in these setting due to the increasing problem of antibacterial resistance among GNB. These drugs have been used in real-world settings, where they are reported to show clinical and microbiological success in high-risk hematological patients. However, there is a scarcity of data, and where it is present, it is based mainly on case series and case reports (116–122). Table 3 summarizes the main clinical and microbiological data in these studies.

**Table 3:** Summary of the main clinical and microbiological characteristics of the published case series and case reports on the use of ceftazidime/avibactam and ceftolozane/tazobactam in high-risk hematologic cancer patients.

**References**	**Study design**	**Number of patients**	**Microorganisms mechanisms of resistance**	**Type of infections**	**Combination therapy**	**All-cause 30-day case-fatality rate**	**Recurrence/resistance development**	**Comments**
**Ceftazidime/Avibactam**								
Castón et al. (120)	Multicenter, retrospective. Patients treated with C/A were compared with those receiving other active antibiotics	8 C/A vs. 23 other active antibiotics	Carbapenemase-producing *Enterobacterales* (80.6% *K. pneumoniae*) 61.3% OXA-48, 38.7% KPC	Bacteremia	100% Combinations included: aminoglycosides (7), carbapenems (3), fosfomycin (2), tigecyclin (2), and/or colistin (2)	25% with C/A vs. 52% with other active agents	None/None	All treatment regiments were used as targeted therapy 77.4% were neutropenic The number of death events were too small to detect significant differences
Metafuni et al. (121)	Case series of patients presenting with persistent sepsis or septic shock	3	Carbapenemase-producing *K. pneumoniae* (*n* = 2) MDR-*P. aeruginosa* (*n* = 1)	Bacteremia	100% Meropenem (2), tigecyclin (3), colisitn (2)	33%	None/None	All treatment regiments were used as targeted therapy All patients were neutropenic
Hobson et al. (122)	Case report of a pediatric patient	1	NDM-1-producing *Morganella morganii*	Bacteremia	Aztreonam (ATM)	0	None/None	The patient was neutropenic MIC for the combination of ATM + C/A was 0.016 mg/L
**Ceftolozane/Tazobactam**								
Hakki and Lewis (116)	Retrospective case series	6 patients received 7 cycles of C/T	MDR-*P. aeruginosa*	Bacteremia (3), Pneumonia (3), SSTI (1)	None	0	1 case/1 case	Four patients (66.6%) were neutropenic and two (33.3%) were HSCT recipients In two cases C/T was initiated empirically
Fernández-Cruz et al. (117)	Retrospective, case-control. Patients treated with C/T were compared with those receiving other active antibiotics	19 C/T vs. 38 other active antibiotics	*P. aeruginosa* (51.2% were MDR)	Primary bacteremia (4) Pneumonia (5), perianal infection (3), UTI (2), SSTI (1)	42% Amikacin + levofloxacin (2), amikacin (4), colistin (1), and fosfomycin (1)	5.3% with C/T vs. 28.9% with other active agents	3 cases/None	C/T was used empirically in 3 cases, and as targeted 6 patients had secondary bacteremia >60% were neutropenic
Aitken et al. (118)	Case report of a pediatric patient	1	MDR-*P. aeruginosa*	Bacteremia	Tobramycin and ciprofloxacin	0	None/1	The patient was neutropenic The MIC to C/T increased from to 6 to 8 μg/mL
So et al. (119)	Case report	1	MDR-P. aeruginosa	Bacteremia	Tobramycin	0	None/1	The patient was neutropenic Bacteremia cleared with the combination of a pharmacodynamically driven dose of C/T and tobramycin with resultant synergy

*MDR, multidrug-resistant; SSTI, skin and soft tissue infection; HSCT, hematopoietic stem cell transplant; UTI, urinary tract infection*.

An alternative strategy for the treatment of infections due to resistant GNB organisms is to use existing BLAs by extended or continuous infusion to maximize their pharmacokinetic (PK) activity (123). Critically ill patients present certain physiopathologic changes, mainly due to an increased volume of distribution and an increased renal clearance, making them excellent targets for this strategy. In fact, two meta-analyses have shown an association between prolonged BLA infusions and lower mortality rates in critically ill patients (124, 125). Cancer patients with FN may be considered similar to critically ill patients in terms of the intra- and inter-individual variability of PK parameters (126–128).

Regarding the clinical impact of optimizing the use of BLAs, data are limited for neutropenic patients and restricted to certain antibiotics (129, 130). A recent single-center randomized clinical trial found that extended infusion with a BLA was associated with superior outcomes than intermittent infusion, the greatest benefit observable in patients with pneumonia (131). Nevertheless, that study had some methodological limitations, and there were no PK studies to support the clinical results (132). Currently, a multicenter, open label, randomized, superiority clinical trial is being performed (EudraCT 2018-001476-37, ClinicalTrials.gov: NCT04233996) to assess the clinical efficacy of extended BLA infusions in hematological patients with neutropenia. Other secondary outcomes include PK/pharmacodynamic target achievement, bacteremia clearance, CRP decrease, overall 30-day case-fatality rate, and adverse events. Finally, a population PK model of the BLA studied will be developed (133).

In the current era of emerging antimicrobial resistance, antimicrobial stewardship is of paramount importance in order to decrease the overall antimicrobial consumption and hinder resistance dissemination. In this regard, the last guidelines recommend applying antimicrobial stewardship strategies in patients with sepsis and septic shock, such as de-escalation and/or discontinuation of antibiotic within the first few days in response to clinical improvement and infection resolution and/or lack of evidence of infection (95). Cancer patients with sepsis and septic shock can be probably managed in the same way. In this line, we recently published a randomized clinical trial involving high-risk hematologic patients with febrile neutropenia without microbiologically documented infection, in which we demonstrated that empirical antibiotic therapy can be discontinued after 72 h of apyrexia and clinical recovery irrespective of the neutrophil count (134). This clinical approach showed to reduce unnecessary exposure to antimicrobials and to be safe.

### ICU Management

The total number of patients with cancer who need ICU admission has increased dramatically over time, presently accounting for up to 15% of all admissions (8, 27–30). Overall mortality has also decreased and survivors achieve remission and quality of life after ICU admission, similar to non-ICU patients (37, 135). Some important changes in the management of critically ill cancer patients over recent decades have influenced these improved outcomes. These include the following:

a) Changes in ICU admission policies that may have favored faster admission of more candidates, leading to early treatment of organ dysfunction (37, 136).
b) Many so-called classic predictors of mortality (e.g., neutropenia, underlying disease, blood transfusion requirements, and second-line therapies) are no longer relevant or influence the therapeutic approach less (32, 137, 138).
c) Improved collaboration between hematologists/oncologists and intensive care providers (137–140).
d) Improved management of sepsis in neutropenic patients, including escalation and de-escalation strategies for antimicrobial therapy, source control (e.g., catheter removal), and/or conducting surgery if indicated regardless of the presence of cytopenias (141–143).
e) Using specific therapies for selected patients with hematological cancers in ICU has also proven to be feasible and associated with significant survival benefit (144–147).

## Current Gaps and Future Research

Although much progress has been made in the understanding and management of sepsis in cancer patients, much is still to be done.

The increasing number of cancer patients who potentially require ICU management will necessitate a comprehensive revision of ICU admission policies. Early recognition of sepsis based on routine clinical, biochemical, and radiological signs is still inaccurate in cancer. Future diagnostic strategies must therefore incorporate newer tests with improved diagnostic performances, easier non-invasive sampling, and shorter response time. None of the currently available biomarkers have demonstrated sufficient sensitivity and/or specificity for use in clinical practice. Identifying new biomarkers reflecting host response and/or pathogen invasion may allow better differentiation of infectious from non-infectious processes and the early and safe discontinuation of antimicrobial therapy.

In addition, gaining a better understanding of the mutual interaction between cancer and sepsis, as well as the alterations in innate and adaptive immune cell functions, could lead to the development of potential therapeutic interventions. Identifying biomarkers that can accurately detect and quantify immune suppression in cancer patients with sepsis will be key to the design of immunomodulatory therapeutic strategies.

The adequacy of IEAT should be improved in cancer patients with sepsis, and efforts should be made to ensure adherence to current guidelines, with adaptation to local epidemiology where necessary. Studies should also continue to clarify the role of new antibiotics, such as ceftolozane/tazobactam and ceftazidime/avibactam, in cancer patients with sepsis, particularly when used empirically. Other novel antibiotics displaying activity against MDR-GNB are currently under clinical evaluation (e.g., imipenem/relabactam, plazomicin, cefiderocol, meropenem/vaborbactam, and eravacycline), and given time, these may improve the antibiotic armamentarium.

Finally, combining knowledge of more rigorous and thorough patient stratification and selection, strategic and careful long-term monitoring of immune function, and targeted immunomodulatory treatment could optimize clinical benefits for surviving initial sepsis.

## Author Contributions

CG and JC were responsible for the study conception and design. CG, AA-P, and GC performed the literature review. CG drafted the manuscript. All authors approved the final version of the manuscript.

## Conflict of Interest

CG has served as speaker at scientific meetings sponsored by Pfizer and MSD. GC has participated as speaker at scientific meetings sponsored by Pfizer. JC has participated as speaker at scientific meetings sponsored by Pfizer and MSD. The remaining author declares that the research was conducted in the absence of any commercial or financial relationships that could be construed as a potential conflict of interest.
